# Serology for the diagnosis of human hepatic cystic echinococcosis and its relation with cyst staging: A systematic review of the literature with meta-analysis

**DOI:** 10.1371/journal.pntd.0009370

**Published:** 2021-04-28

**Authors:** Francesca Tamarozzi, Ronaldo Silva, Veronica Andrea Fittipaldo, Dora Buonfrate, Bruno Gottstein, Mar Siles-Lucas

**Affiliations:** 1 Department of Infectious-Tropical Diseases and Microbiology, IRCCS Sacro Cuore Don Calabria Hospital, Negrar, Verona, Italy; 2 Institute of Infectious Diseases, Faculty of Medicine, University of Bern, Bern, Switzerland; 3 Instituto de Recursos Naturales y Agrobiología de Salamanca (IRNASA-CSIC), Salamanca, Spain; Imperial College London, Faculty of Medicine, School of Public Health, UNITED KINGDOM

## Abstract

**Background:**

The diagnosis of cystic echinococcosis (CE) is primarily based on imaging, while serology should be applied when imaging is inconclusive. CE cyst stage has been reported among the most important factors influencing the outcome of serodiagnosis. We performed a systematic review and meta-analysis of the relation between cyst stage of hepatic CE and diagnostic sensitivity of serological tests, to evaluate whether their relation is a consistent finding and provide guidance for the interpretation of results of serological tests.

**Methodology/Principal findings:**

MEDLINE, EMBASE, CENTRAL, and Lilacs databases were searched on December 1^st^ 2019. Original studies published after 2003 (year of publication of the CE cyst classification), reporting sensitivity of serological tests applied to the diagnosis of human hepatic CE, as diagnosed and staged by imaging, were included. The quality of studies was assessed using the Newcastle-Ottawa Scale. Data from 14 studies were included in the meta-analysis. Summary estimates of sensitivities and 95% confidence intervals were obtained using random effects meta-analysis. Overall, test sensitivity was highest in the presence of CE2 and CE3 (CE3a and/or CE3b), and lowest in the presence of CE5 and CE4 cysts. ELISA, ICT and WB showed the highest sensitivities, while IHA performed worst.

**Conclusions/Significance:**

The results of our study confirm the presence of a clear and consistent relation between cyst stage and serological tests results. Limitations of evidence included the heterogeneity of the antigenic preparations used, which prevented to determine whether the relation between cyst stage and sensitivity was influenced by the type of antigenic preparation, the paucity of studies testing the same panel of sera with different assays, and the lack of studies assessing the performance of the same assay in both field and hospital-based settings. Our results indicate the absolute need to consider cyst staging when evaluating serological results of patients with hepatic CE.

## Author summary

Cystic echinococcosis is a neglected zoonosis induced by the development of parasitic cysts in intermediate hosts, including humans, mostly in the liver. The diagnosis of CE is based on imaging. As CE cysts may assume different aspects (stages), the range of differential diagnoses is broad, from harmless simple cysts to neoplasms. Serological assays for the detection of serum antibodies are applied when imaging is inconclusive, but their performance depend on a number of factors, among which cyst stage has been reported as important. If this was a robust finding, it would be absolutely required to interpret serological findings in the light of CE cyst staging. The results of our systematic review and meta-analysis of the relation between cyst stage of hepatic CE and diagnostic sensitivity of serological tests confirmed that such relation is clear and consistent, and indicate the absolute need to consider cyst staging when evaluating serology results of patients with hepatic CE.

## Introduction

Cystic echinococcosis (CE) is a neglected zoonosis caused by infection with the larval stage of the cestode *Echinococcus granulosus sensu lato* [1]. The parasite is transmitted between canid definitive hosts and livestock, mainly sheep, as intermediate hosts [2]. Humans are dead-end intermediate hosts, in whom the larval stage develops as fluid-filled cysts mainly in liver and lungs [3]. It has been estimated that around 1 million Disability-Adjusted Life Years are lost due to human CE [4], but the prevalence and number of infected people, on which to base disease burden calculations, are difficult to quantify. Hampering the implementation of comprehensive population-wide studies on CE are in part the peculiar socio-epidemiological features of the infection [5], and in part the fact that current diagnostic tools are not suitable for an efficient mapping of infection distribution at population level [6]. This, however, is one of the critical actions indicated by the WHO for echinococcosis in the recently issued “2021–2030 road map for neglected tropical diseases” [7]. In the same document, to “*define target product profile and develop optimal diagnostic for humans*” is indicated as a critical action required, in order to reach disease-specific targets. Furthermore, the diagnosis of CE is often difficult also in the clinical setting [3,8–10].

The primary diagnosis of CE is based on imaging, while serology for the detection of circulating serum antibodies is a complementary tool [8]; at present no circulating antigen-detection test is commercially available. Ultrasonography (US) is the reference technique for the diagnosis and characterization of CE cysts in US-accessible organs [8,11], especially the liver, which is the affected organ in >70% of cases. Along their evolution, CE cysts change in structure and appearance on imaging, and these stages are currently classified by the WHO Informal Working Group on Echinococcosis (WHO-IWGE) classification [8,12]. This classification is also important to guide the clinical management of infected patients [8,13]. The availability of portable, relatively cheap US machines has offered the possibility to apply this technique as a point-of-care exam also in remote rural areas. However, the lack of instruments and expertise is still an issue in many endemic countries.

Clearly, a robust and accurate serological assay, e.g. in the format of a rapid diagnostic test, would allow more easily the implementation of population-based epidemiological studies. Unfortunately, currently available serodiagnostic tests for CE are not standardized and their performances are not suitable for application in population-based studies [6,9]. Furthermore, even in the clinical setting, serology should be applied only after a lesion suspect of CE is visualized on imaging, and its results should be interpreted with caution [8,10]. Test-related, setting-related (prevalence/pre-test probability), and cyst-related factors influence the outcome and interpretation of serodiagnostic tests for CE. Among the latter, several studies found that sensitivity was higher in cases of hepatic as compared to extra-hepatic localization, in case of multiple and large cysts, and in case of a recent pharmacological and/or interventional treatment; but the most consistent factor associated with serological results for hepatic CE has been reported to be the cyst stage [14,15]. In this light, the distribution of cyst stages in a population, as well as the definition of cyst stage for the differential diagnosis of evocative lesions at an individual patient’s level is crucial for the interpretation of serological results. On the other hand, knowing how serological tests perform in relation to different cyst stages is crucial, but seldom investigated and applied.

We performed a systematic review and meta-analysis of the relation between cyst stage of liver CE and diagnostic sensitivity of serological tests, to provide an overall guidance for the interpretation of results of serological tests for the diagnosis of hepatic CE.

## Methods

### Search strategy

The review protocol was submitted to PROSPERO international prospective register of systematic reviews (Registration Number CRD420201656630). MEDLINE (PubMed), EMBASE, CENTRAL (Cochrane Library), and Lilacs (Bireme) databases were searched on December 1^st^ 2019. The databases were searched using database-specific strings based on the following keywords: *Echinococcus granulosus*, *Echinococcus* infection, echinococcosis, cystic echinococcosis, hydatidosis, hydatid cyst, hydatid disease, serology, serological test, serological investigations, serodiagnosis. The detailed strategy is available in S1 Text. No language restriction was applied. Merged search results were screened for potentially relevant publication, based on title and abstract, after removal of duplicates. The reference list of all potentially eligible studies and of the review papers were searched for other potentially eligible studies. Eligible papers were restricted to those published from 2003 onwards, i.e. following the publication of the first WHO-IWGE ultrasound cyst classification. The work is presented according to Preferred Reporting Items for Systematic Reviews and Meta-Analyses (PRISMA) recommendations [16] (S1 PRISMA Checklist).

### Inclusion/exclusion criteria, study selection, and data extraction

Original studies reporting sensitivity of serological tests applied to the diagnosis of human CE were included in the review. No restriction was applied regarding publication type (research paper or conference report) or setting (field or clinical setting). Two authors (FT and MSL) independently selected the studies for inclusion in the systematic review, first on the basis of title and abstract, and then of the full text, if retrieved. The selection procedure was carried out using EndNote X7.7 software (Thomson Reuters, Toronto, Ontario, Canada).

Inclusion criteria were: i) presenting original data; ii) cross-sectional, cohort, and case-control diagnostic accuracy study type (i.e. case reports were excluded); iii) including cases with at least one hepatic CE cyst assessed by imaging and staged (or stageable) according to the WHO-IWGE ultrasound classification (CE1, CE2, CE3, CE3a CE3b, CE4, CE5 [8]); v) reporting results (positive/negative) and characteristics of anti-echinococcal antibody assay(s).

Potentially eligible studies were excluded if: i) full text and abstract were both unavailable or only abstract was available but did not convey the needed data or data were not extractable for analysis; ii) diagnosis was not based on imaging; iii) staging was not performed/not reported and/or staging could not be defined based on reported information or images; iv) information regarding the serological assay were not provided or the diagnostic test was not eligible; v) CE cysts localization was extra-hepatic or not specified; vi) study duplication.

The same two authors independently performed the data extraction using a pre-designed data extraction form (Excel file). Data extracted were: i) study type; ii) treatment status of the tested patients; iii) number of patients tested per each CE cyst stage; iv) number of positive individuals per each CE cyst stage; v) serological test(s) characteristics (test format, antigenic preparation).

At all review steps, a third author (DB) was in charge of facilitating discussion and reaching consensus in case of disagreement between the reviewers.

### Quality assessment

An adapted version of the Newcastle-Ottawa Scale (NOS) for assessing the quality of observational studies was applied [17]. The quality of each included paper was assessed in three domains (selection, comparability, outcome). S2 Text summarizes the items included in each domain and the characteristics for the attribution of the “stars” (scoring system). Studies scoring 1 to 3 stars were ranked as “low quality”; 4 or 5 stars as “high quality”, and 6 or 7 stars as “very high quality”.

### Data analysis

Collected data from included studies were aggregated, stratified by type of test or setting, to obtain summary estimates of sensitivities and 95% confidence intervals (CI) using random effects meta-analysis. The analysis was carried out both including all studies and excluding low-quality studies. DerSimonian and Laird method [18] was used for parameters estimations with the estimate of heterogeneity, measured by the I^2^ statistic, obtained from the Mantel-Haenszel model [19]. Meta-analyses were performed using *metan* package from STATA software version 14 (StataCorp. 2015. Stata Statistical Software: Release 14. College Station, TX: StataCorp LP.). SAS software version 9.4 (SAS Institute, Inc., Cary, NC, USA) and GraphPad-Prism8 (GraphPad Software, San Diego, CA, USA) were used for forest plots and bar graphs respectively. Statistical significance level was fixed at 0.05.

## Results

### Bibliographic search

**Fig 1:**
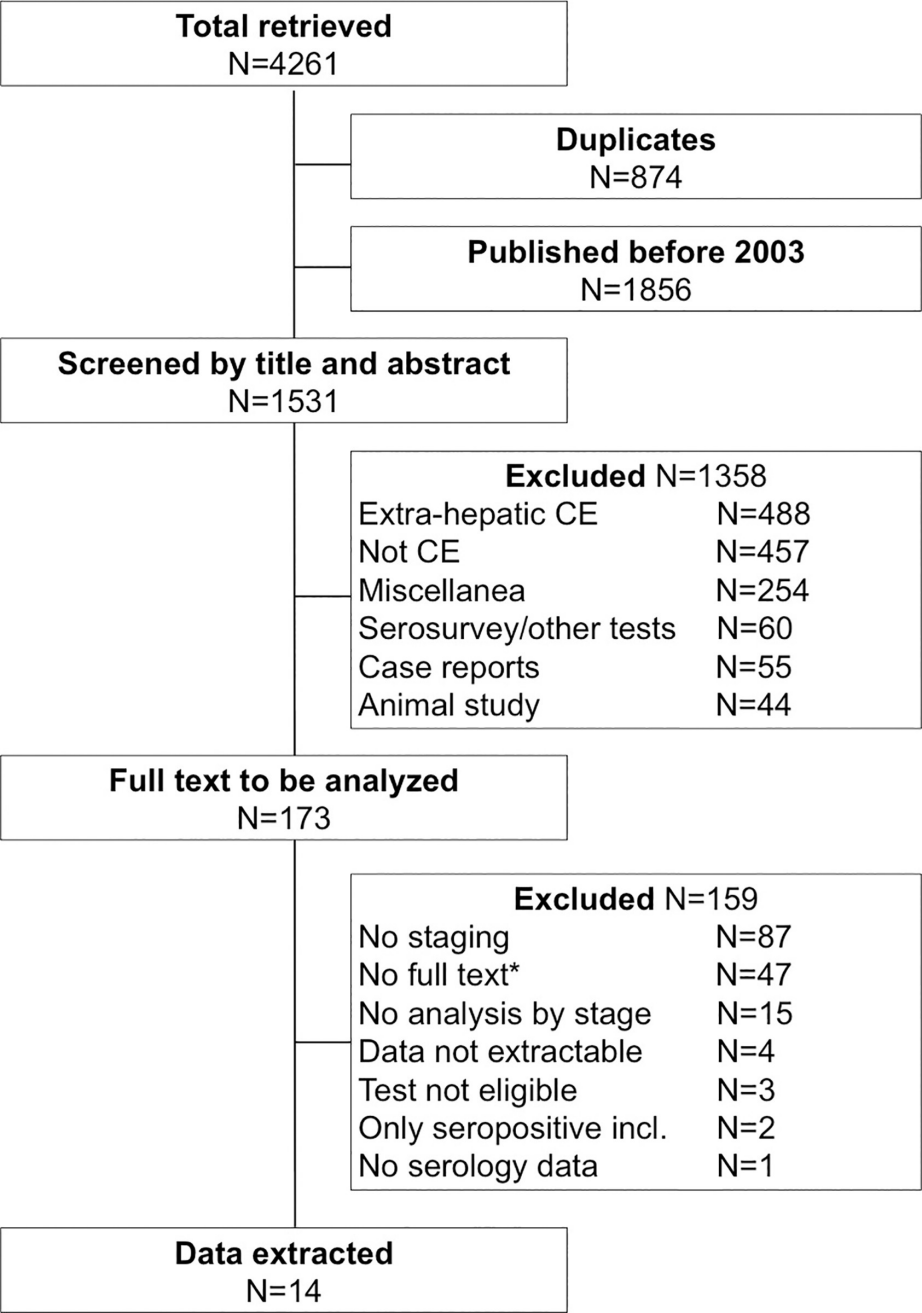
Flow diagram of the literature search.

The literature search and selection of studies to be included are schematized in Fig 1. The databases search retrieved a total of 4261 records, leaving 3387 records after duplicates were removed. A total of 1531 records published after year 2003 were potentially eligible based on title and abstract. Upon evaluation of the full text, 14 articles were selected from which data were extracted (Table 1). The quality assessment of each item included in the three NOS domains is shown in S1 Table.

**Table 1:** Main characteristics of the papers included in the meta-analysis.

Publication	Setting	Study design	Data analysis only of untreated patients possible	Evaluated serology assay format* and number	Quality§
Tiaoying, L. 2005 [21]	Field	Cross-sectional	Yes	1 ELISA	Low
Yang, Y. 2007 [22]	Field	Cross-sectional	Yes	1 ELISA	Low
Li, T. 2011 [23]	Field	Cohort	Yes	1 ELISA	High
Schweiger, A. 2012 [24]	Laboratory	Diagnostic accuracy	No	1 ELISA, 1 EITB	Low
Hernandez-Gonzalez, A. 2012 [25]	Laboratory	Diagnostic accuracy	No	3 ELISA, 1 IHA	High
Tamarozzi, F. 2013 [26]	Laboratory	Diagnostic accuracy	Yes	2 ELISA	High
Piccoli, L. 2014 [27]	Hospital	Cohort	Yes	1 ELISA	High
Lissandrin, R. 2016 [15]	Laboratory	Diagnostic accuracy	Yes	1 ELISA	Very high
Tamarozzi, F. 2016 [28]	Laboratory	Diagnostic accuracy	No	1 ELISA, 3 ICT	Very high
Vola, A. 2018 [20]	Laboratory	Diagnostic accuracy	Yes	2 ICT	High
Hernandez-Gonzalez, A. 2018 [14]	Laboratory	Diagnostic accuracy	No	3 ELISA, 2 ICT	High
Pagnozzi, D. 2018 [29]	Laboratory	Diagnostic accuracy	No	2 ELISA	Low
Han, X. 2019 [30]	Field	Cross-sectional	Yes	5 ELISA	Very high
Vola, A. 2019 [10]	Hospital	Cohort	Yes	1 ELISA, 1 IHA, 1 WB	Very high

*ELISA = enzyme-linked immunosorbent assay; EITB = enzyme-linked immunoelectrotransfer blot; IHA = indirect hemagglutination; ICT = immunochromatographic test. WB = western blot. EITB and WB are basically identical methods and are analyzed together.

§Based on an adapted version of the Newcastle-Ottawa Scale for assessing the quality of observational studies (S2 Text and S1 PRISMA Checklist).

The selected studies were divided into three groups, depending on the setting: field studies for CE cases detected by imaging in population survey campaigns, hospital studies for CE cases detected by imaging in the clinical setting and analysed as cohorts, and laboratory studies for CE cases detected in clinical setting and including a control group for test accuracy evaluation. The majority (11/14) of the evaluated studies included seroassays detecting total IgG, in one case (Vola et al 2018 [20]) the study included two serological tests, one detecting IgG and one detecting IgG4, and in two studies (Tiaoying et al 2005; Yang et al 2007 [21,22]) the antibody isotype detected was not specified. The antigenic preparations used in the seroassays included hydatid cyst fluid, purified native antigens, recombinant antigens, synthetic antigens, and their variable combinations. The extraction data sheet is available as S2 Table.

### Results of analysis concerning only untreated cysts, in all settings

**Fig 2:**
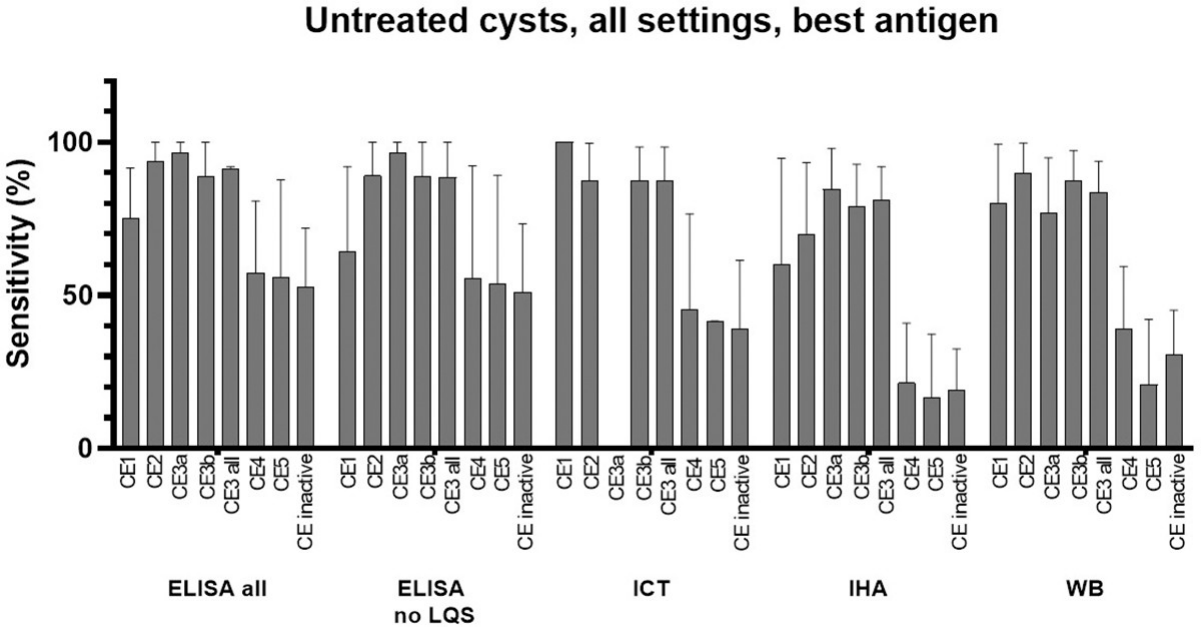
Sensitivity (%) of different serology tests for the diagnosis of untreated hepatic CE cysts according to cyst stage. “CE3 all” = data from CE3a + CE3b + CE3 not divided into CE3a and CE3b. “CE inactive” = data from CE4 + CE5 + CE4 and CE5 not divided into CE4 and CE5. LQS = Low Quality Studies. Data from papers referring to the field or to the hospital/lab setting were included. In case more than one assay per type was investigated in the same paper (e.g. more than one ELISA test), the one with the best sensitivity was used for the meta-analysis. Error bars represent 95% CI.

First, we analyzed the sensitivity of diagnostic tests applied to samples of patients untreated for CE, irrespective of the study setting. In cases where samples were tested using more than one assay of the same format, the assay with the best sensitivity was included in the meta-analysis. Fig 2 summarizes the estimated sensitivity of ELISA, ICT, IHA, and WB assays by cyst stage. The Forest plots of the analyses are presented in S1 Fig. Overall, and granted the higher number of studies using ELISA as compared to other assay formats, tests sensitivity was highest in the presence of CE2 and CE3 (CE3a and/or CE3b), and lowest in the presence of CE5 and CE4 cysts. This trend was maintained when the analysis of ELISA was carried out without low quality studies. Among tests, ELISA and ICT showed the highest sensitivities, while IHA performed worst. The summary of results for ELISA assays is shown in Table 2.

**Table 2:** Estimated sensitivity (Se %) with 95%CI of ELISA test with best performance.

Cyst stage	Only untreated cysts							Treated and untreated cysts	
	All settings		Field setting		Laboratory + hospital setting Se% (95%CI)	Laboratory setting Se% (95%CI)	Hospital setting Se% (95%CI)	All settings	
	All studies Se% (95%CI)	No low-quality studies Se% (95%CI)	All studies Se% (95%CI)	No low-quality studies Se% (95%CI)				All studies Se% (95%CI)	No low-quality studies Se% (95%CI)
**CE1**	75 (58–92)	64 (36–92)	85 (71–100)	75 (38–100)	50 (23–76)	57 (18–90)^	40 (5–85)^	82 (71–92)	79 (64–93)
**CE2**	94 (85–100)	89 (78–100)	95 (74–100)	95 (74–100)^	83 (67–98)	88 (62–98)^	70 (35–97)^	97 (92–100)	94 (88–100)
**CE3a**	97 (85–100)	97 (85–100)°	-	-	-	-	-	99 (94–100)	99 (94–100)°
**CE3b**	89 (65–100)	89 (65–100)°	-	-	-	-	-	93 (87–100)	93 (87–100)°
CE3*	91 (82–92)	87 (75–100)	99 (95–100)	100 (88–100)^	82 (74–90)	82 (71–91)^	81 (65–92)^	92 (87–98)	91 (85–98)
**CE4**	57 (34–81)	56 (19–92)	71 (47–96)	91 (71–99)^	38 (26–50)	40 (25–56)^	36 (19–56)^	60 (42–77)	59 (36–82)
**CE5**	56 (24–88)	54 (18–89)	73 (36–100)	83 (62–100)	24 (1–47)	14 (5–29)^	38 (19–59)^	53 (30–75)	48 (24–73)
**Inactive**§	53 (34–72)	51 (29–73)	73 (50–97)	90 (75–99)	34 (25–43)	36 (17–56)	33 (22–43)	53 (38–69)	51 (33–69)

*CE3 = CE3a+CE3b+CE3 not divided into the two sub-stages.

^§^Inactive = CE4+CE5+CE4/5 inactive stages not divided into the two sub-stages.

°The papers scored as low quality were not investigating this group stage.

^Only one paper included.

### Analysis of ELISA results regarding untreated cysts, per study setting

**Fig 3:**
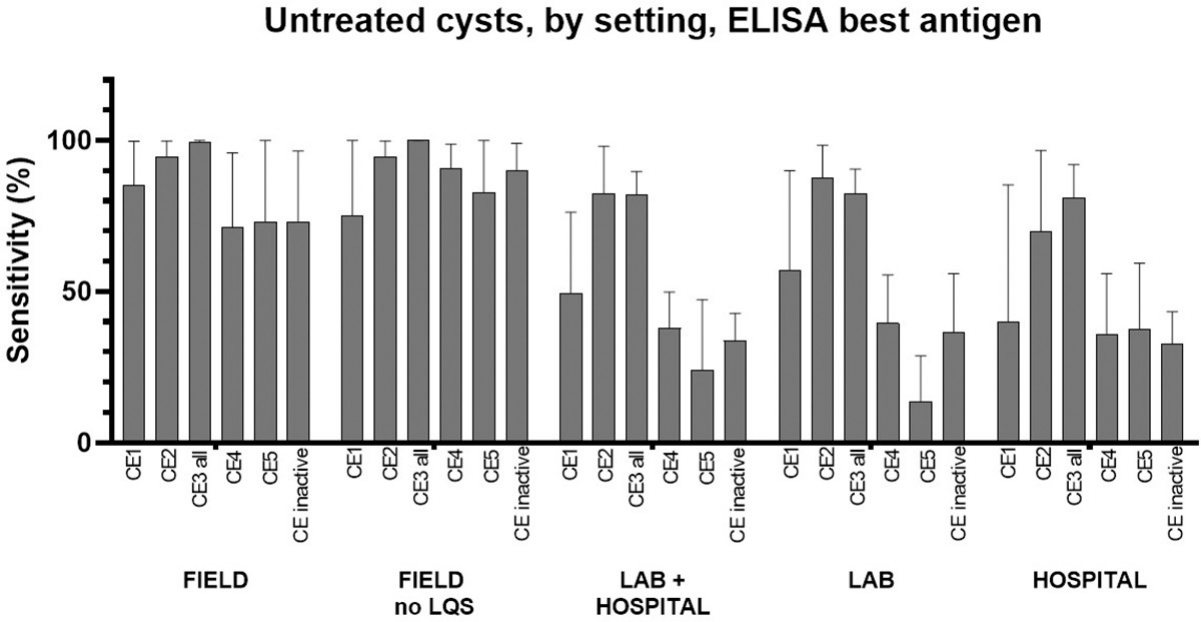
Sensitivity (%) of ELISA tests for the diagnosis of untreated hepatic CE cysts according to cyst stage and setting (field vs lab and/or hospital). “CE3 all” = data from CE3a + CE3b + CE3 not divided into CE3a and CE3b. “CE inactive” = data from CE4 + CE5 + CE4 and CE5 not divided into CE4 and CE5. LQS = Low Quality Studies. In case more than one assay per type was investigated in the same paper (e.g. more than one ELISA test), the one with the best sensitivity was used for the meta-analysis. Error bars represent 95% CI.

The setting in which the evaluation of serological tests is carried out may influence test results as it may favor the inclusion of patients with particular characteristics, and in turn influence the probability of a test being seropositive. For example, it is conceivable that untreated patients accessing the hospital setting may be more often symptomatic than subject enrolled in population-based field studies, possibly due to cyst characteristics (e.g. size, loss of integrity of the cyst wall) which in turn influence test performance. We therefore evaluated the sensitivity of ELISAs grouped by study setting (field, laboratory + hospital, laboratory, and hospital). In cases where samples were tested by using more than one ELISA, the assay with the best sensitivity was included in the meta-analysis. Results are shown in Fig 3 and Table 2. The Forest plots of the analyses are presented in S2 Fig. Even divided by setting, sensitivity results mirrored those of the previous general analysis, with highest sensitivities observed for CE2 and CE3 (CE3a and CE3b) cysts and lowest sensitivities for CE5 cysts. Interestingly, higher sensitivities were reported by studies applied in the field compared to the laboratory and/or hospital setting.

### Analysis of tests results regarding cysts independently of treatment, in all settings

**Fig 4:**
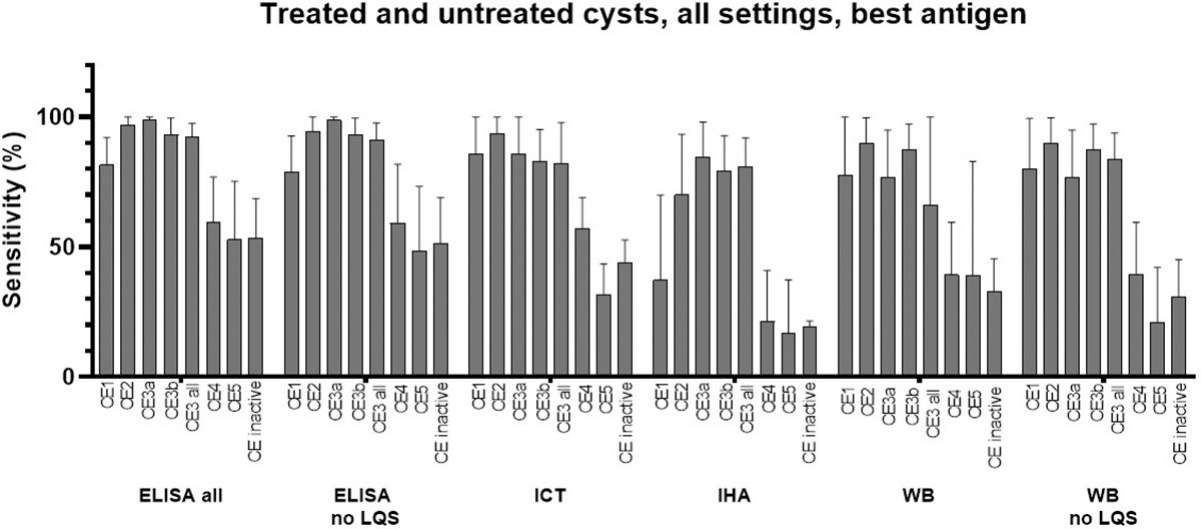
Sensitivity (%) of ELISA tests for the diagnosis of hepatic CE cysts, irrespective of previous treatment and setting. “CE3 all” = data from CE3a + CE3b + CE3 not divided into CE3a and CE3b. “CE inactive” = data from CE4 + CE5 + CE4 and CE5 not divided into CE4 and CE5. LQS = Low Quality Studies. In case more than one assay per type was investigated in the same paper (e.g. more than one ELISA test), the one with the best sensitivity was used for the meta-analysis. Error bars represent 95% CI.

Often the putative previous treatment status of a patient is not known, or uncertain. Therefore, we investigated the sensitivity of diagnostic tests independently from the study setting and treatment status of the patients included in the cohorts. Fig 4 summarizes the estimated sensitivity of ELISA, ICT, IHA, and WB by cyst stage. In the cases where samples were tested by more than one ELISA, the assay with the best sensitivity was included in the meta-analysis. The Forest plots of the analyses are presented in S3 Fig. Sensitivity results by cyst stage and type of test mirrored, and were overall comparable with those obtained by the analysis of samples only from untreated subjects. The summary of results for ELISA is shown in Table 2.

**Fig 5:**
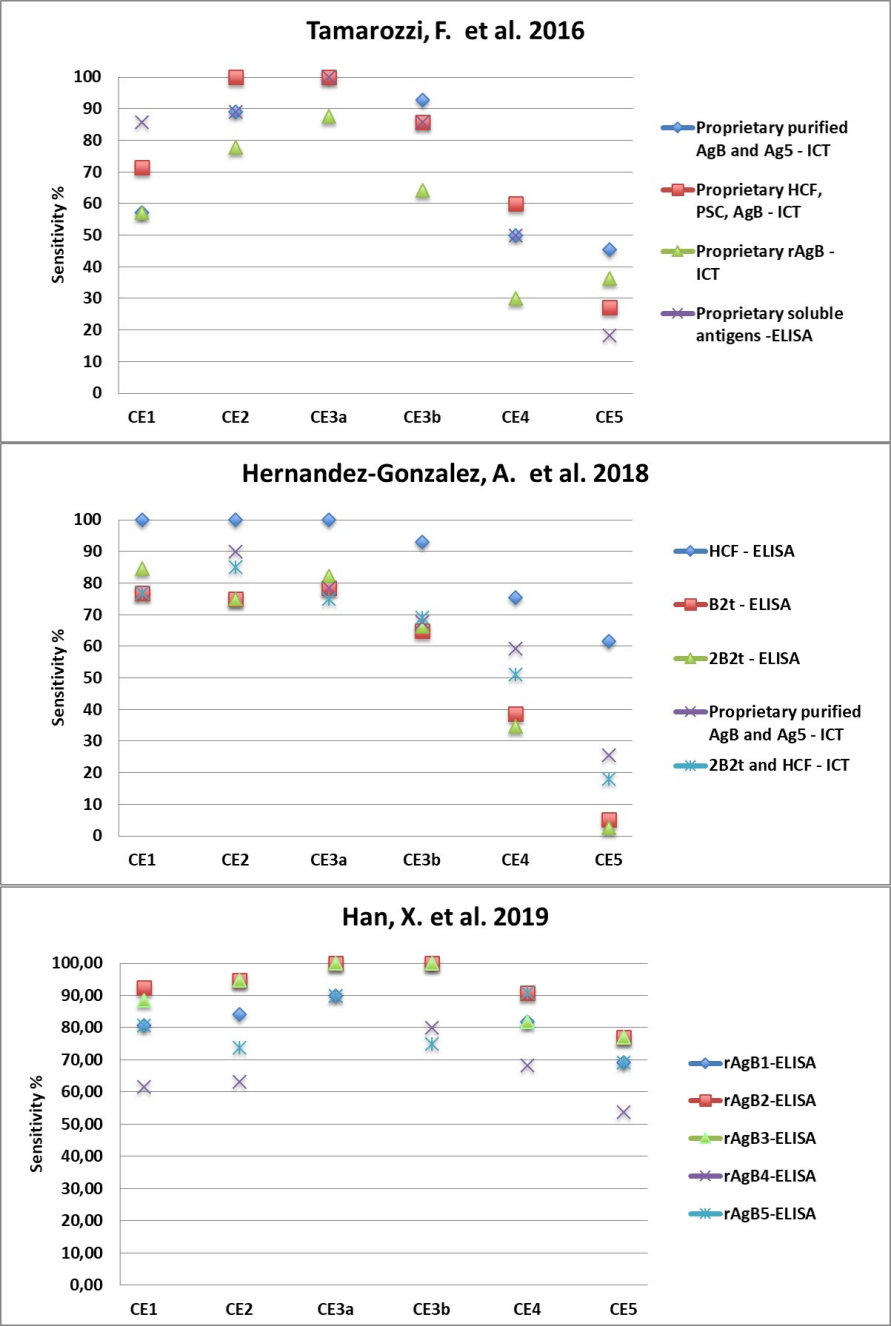
Sensitivity (%) of different assays using a variety of antigenic preparations, as reported by three example recent studies, showing the same pattern according to cyst stage.

Due to the limited number of eligible studies, based on extremely heterogeneous antigenic preparations, it was not possible to determine whether the relation between cyst stage and sensitivity was influenced by the type of antigenic preparation used in the assays. However, studies using different antigenic preparations to test the same panel of sera, although obtaining different values of sensitivity, reported the same pattern of seropositivity according to cyst stage (Fig 5).

## Discussion

Ultrasonography is the tool of reference for diagnosing abdominal CE, both for population screening and individual diagnosis. Moreover, US-based cyst staging is pivotal for clinical decision-making in the presence of uncomplicated hepatic CE cysts [8,13], which is the most common presentation of CE in a population. However, good quality US machines and/or specific expertise on the recognition of pathognomonic CE characteristics are not widely available. Serology is used to support imaging in doubtful cases, while currently available serological tests are not suitable, alone, for application in population-based prevalence studies [6,9]. Also in the clinical setting, the correct interpretation of serological results may be challenging [10]. Several factors, among which CE cyst stage is prominent, have been reported to influence the outcome of serological tests, resulting in a variable rate of false negative results [14,15]. Unfortunately, however, cyst stages are infrequently reported and taken into account when results of serology are evaluated in both everyday clinical practice and in scientific works. Of the 173 potentially eligible studies retrieved in this systematic review, 87 (50%) were excluded because they did not describe cyst staging.

We performed this systematic review and meta-analysis with the aims of (i) evaluating whether the relation between cyst stage of hepatic CE and sensitivity of serology test was a robust finding, and (ii) to provide an overall guidance for the interpretation of results of serology for the diagnosis of hepatic CE. The results of our study confirm the presence of a clear relation between cyst stage and serology tests results, independently of the seroassay format, study setting, and inclusion of samples from only untreated or both treated and untreated patients. Unfortunately, due to the heterogeneity of the antigenic preparations described in the included papers, it was not possible to formally assess whether this relation was influenced by the type of antigenic preparation. However, in agreement with a previous study [15], this seems not the case, thus the relation between seropositivity rate and cyst stage, with the highest values in the presence of CE2 and CE3 (generally CE3a>CE3b), and lowest in the presence of CE5 and CE4 cysts, appears a robust finding.

Overall, our results indicate that ELISA and ICT were the most sensitive test formats, followed by WB, while IHA had the lowest sensitivity. However, this result should be taken with caution as only one study included in this study [10] investigated different test formats using the same, comprehensive panel of sera, finding that WB had the highest sensitivity while IHA and ELISA were comparable.

Finally, we observed that sensitivity was apparently lower when tests were applied in diagnostic accuracy and hospital-based cohorts compared to the field setting. This finding was unexpected, as intuitively one would have forecasted that untreated patients accessing the hospital setting might be more often symptomatic, and therefore had cysts with characteristics (e.g. large size, loss of integrity of the cyst wall) that are positively correlated with seropositivity. Furthermore, three of the four field-based studies used tests based on recombinant antigens, which are known to yield a lower sensitivity when compared to native antigens. It must be noted, however, that no study evaluated in this review investigated the same test in both field and clinical setting, making also this observation to be taken with caution. In this regard, a study by Gavidia et al. [31] describing an US-based survey for CE coupled with two WB assays, one containing hydatid cyst fluid and the other a recombinant antigen (rEpC1-GST), showed that the WB based on rEpC1-GST had a sensitivity of 16.7% when samples from all individuals with hepatic CE were included, and of 29.4% when calcified cysts were excluded. The same recombinant antigen was tested on sera from 324 surgically confirmed CE patients (of which 116 obtained before surgery), yielding an overall sensitivity of 92.2% [32]. Thus, the unusually high sensitivity reported for serology in the field assays analyzed in this review remains difficult to explain, and may also derive from the limited number of studies included in the analysis. Furthermore, other factors that influence serology results, such as cysts number and size [14,15], were not analyzed in this work; these factors, which may have had a different distribution in the patient cohorts of the papers included in this analysis, might have influenced the results of our study. However, these results highlight the need to evaluate all serodiagnostic tests in different settings and using multiple cohorts, to obtain a more comprehensive assessment of their performances.

In conclusion, the results of our systematic review and meta-analysis, although based on a limited number of eligible studies, show that a consistent pattern exists between the cyst stage of hepatic CE and serological tests results, independently of the seroassay format, study setting, and inclusion of samples from only untreated or both treated and untreated patients, with the highest sensitivity obtained in the presence of CE2 and CE3 and the lowest in the presence of inactive cysts. These results indicate the absolute need to describe and take into account cyst staging when evaluating serological results of patients with hepatic CE, in both the clinical setting and in research work.

## Supporting information

S1 PRISMA Checklist (DOC)

S1 Text Literature search strategy. (DOCX)

S2 Text Adapted Newcastle-Ottawa Scale for study quality assessment. (DOCX)

S1 Table Quality assessment of the included studies. (DOCX)

S2 Table Data extraction sheet. (XLSX)

S1 Fig Forest plots of sensitivity of CE serology from publications including patients with untreated cysts, in all settings. (TIF)

S2 Fig Forest plots of sensitivity of CE ELISA serology from publications including patients with untreated cysts, according to the study settings. (TIF)

S3 Fig Forest plots of sensitivity of CE serology from publications including patients with untreated and treated cysts, in all settings. (TIF)

## References

[1] Thompson RC. Biology and systematics of Echinococcus. Adv Parasitol. 2017;95:65–109. 10.1016/bs.apar.2016.07.001 28131366

[2] Romig T, Deplazes P, Jenkins D, Giraudoux P, Massolo A, Craig PS, et al. Ecology and life cycle patterns of Echinococcus species. Adv Parasitol. 2017;95:213–314. 10.1016/bs.apar.2016.11.002 28131364

[3] Kern P, Menezes da Silva A, Akhan O, Mullhaupt B, Vizcaychipi KA, Budke C, et al. The echinococcoses: diagnosis, clinical management and burden of disease. Adv Parasitol. 2017;96:259–369. 10.1016/bs.apar.2016.09.006 28212790

[4] Budke CM, Deplazes P, Torgerson PR. Global socioeconomic impact of cystic echinococcosis. Emerg Infect Dis. 2006;12:296–303. 10.3201/eid1202.050499 16494758 PMC3373106

[5] Deplazes P, Rinaldi L, Alvarez Rojas CA, Torgerson PR, Harandi MF, Romig T, et al. Global distribution of alveolar and cystic echinococcosis. Adv Parasitol. 2017;95:315–493. 10.1016/bs.apar.2016.11.001 28131365

[6] Torgerson PR, Deplazes P. Echinococcosis: diagnosis and diagnostic interpretation in population studies. Trends Parasitol. 2009;25:164–170. 10.1016/j.pt.2008.12.008 19269248

[7] World health Organization. Ending the neglect to attain the Sustainable Development Goals: a road map for neglected tropical diseases 2021–2030. Geneva: World Health Organization; 2020.

[8] Brunetti E, Kern P, Vuitton DA, Writing Panel for the WHO-IWGE. Expert consensus for the diagnosis and treatment of cystic and alveolar echinococcosis in humans. Acta Trop. 2010;114:1–16. 10.1016/j.actatropica.2009.11.001 19931502

[9] Siles-Lucas M, Casulli A, Conraths FJ, Muller N. Laboratory diagnosis of Echinococcus spp. in human patients and infected animals. Adv Parasitol. 2017;96:159–257. 10.1016/bs.apar.2016.09.003 28212789

[10] Vola A, Manciulli T, De Silvestri A, Lissandrin R, Mariconti M, Siles-Lucas M, et al. Diagnostic performances of xommercial ELISA, Indirect Hemagglutination, and Western Blot in differentiation of hepatic echinococcal and non-echinococcal lesions: a retrospective analysis of data from a single referral centre. Am J Trop Med Hyg. 2019;101:1345–1349. 10.4269/ajtmh.19-0556 31674293 PMC6896875

[11] Stojkovic M, Rosenberger K, Kauczor HU, Junghanss T, Hosch W. Diagnosing and staging of cystic echinococcosis: how do CT and MRI perform in comparison to ultrasound? PLoS Negl Trop Dis. 2012;6:e1880. 10.1371/journal.pntd.0001880 23145199 PMC3493391

[12] Rogan MT, Hai WY, Richardson R, Zeyhle E, Craig PS. Hydatid cysts: does every picture tell a story? Trends Parasitol. 2006;22:431–438. 10.1016/j.pt.2006.07.003 16843726

[13] Stojkovic M, Weber TF, Junghanss T. Clinical management of cystic echinococcosis: state of the art and perspectives. Curr Opin Infect Dis. 2018;31:383–392. 10.1097/QCO.0000000000000485 30124496

[14] Hernandez-Gonzalez A, Sanchez-Ovejero C, Manzano-Roman R, Gonzalez Sanchez M, Delgado JM, Pardo-Garcia T, et al. Evaluation of the recombinant antigens B2t and 2B2t, compared with hydatid fluid, in IgG-ELISA and immunostrips for the diagnosis and follow up of CE patients. PLoS Negl Trop Dis. 2018;12:e0006741. 10.1371/journal.pntd.0006741 30188936 PMC6143278

[15] Lissandrin R, Tamarozzi F, Piccoli L, Tinelli C, De Silvestri A, Mariconti M, et al. Factors influencing the serological response in hepatic Echinococcus granulosus infection. Am J Trop Med Hyg. 2016;94:166–171. 10.4269/ajtmh.15-0219 26503271 PMC4710424

[16] https://www.equator-network.org/library/guidance-on-scientific-writing/ Accessed January 2020.

[17] http://www.ohri.ca/programs/clinical_epidemiology/oxford.asp Accessed January 2020.

[18] DerSimonian R, Laird N. Meta-analysis in clinical trials. Control Clin Trials. 1986;7:177–188. 10.1016/0197-2456(86)90046-2 3802833

[19] Mantel N, Haenszel W. Statistical aspects of the analysis of data from retrospective studies of disease. J Natl Cancer Inst. 1959;22:719–748. 13655060

[20] Vola A, Tamarozzi F, Noordin R, Yunus MH, Khanbabaie S, De Silvestri A, et al. Preliminary assessment of the diagnostic performances of a new rapid diagnostic test for the serodiagnosis of human cystic echinococcosis. Diagn Microbiol Infecti Dis. 2018;92:31–33. 10.1016/j.diagmicrobio.2018.04.007 29776711

[21] Tiaoying L, Jiamin Q, Wen Y, Craig PS, Xingwang C, Ning X, et al. Echinococcosis in Tibetan populations, western Sichuan Province, China. Emerg Infect Dis. 2005;11:1866–73. 10.3201/eid1112.050079 16485472 PMC3367622

[22] Yang YR, Craig PS, Ito A, Vuitton DA, Giraudoux P, Sun T, et al. A correlative study of ultrasound with serology in an area in China co-endemic for human alveolar and cystic echinococcosis. Trop Med Int Health. 2007;12:637–646. 10.1111/j.1365-3156.2007.01834.x 17445131

[23] Li T, Ito A, Pengcuo R, Sako Y, Chen X, Qiu D, et al. Post-treatment follow-up study of abdominal cystic echinococcosis in tibetan communities of northwest Sichuan Province, China. PLoS Negl Trop Dis. 2011;5:e1364. 10.1371/journal.pntd.0001364 22039558 PMC3201905

[24] Schweiger A, Grimm F, Tanner I, Mullhaupt B, Bertogg K, Muller N, et al. Serological diagnosis of echinococcosis: the diagnostic potential of native antigens. Infection. 2012;40:139–152. 10.1007/s15010-011-0205-6 22076692

[25] Hernandez-Gonzalez A, Santivanez S, Garcia HH, Rodriguez S, Munoz S, Ramos G, et al. Improved serodiagnosis of cystic echinococcosis using the new recombinant 2B2t antigen. PLoS Negl Trop Dis. 2012;6:e1714. 10.1371/journal.pntd.0001714 22802975 PMC3389031

[26] Tamarozzi F, Sako Y, Ito A, Piccoli L, Grisolia A, Itoh S, et al. Recombinant AgB8/1 ELISA test vs. commercially available IgG ELISA test in the diagnosis of cystic echinococcosis. Parasit Immunol. 2013;35:433–440. 10.1111/pim.12050 23834586

[27] Piccoli L, Tamarozzi F, Cattaneo F, Mariconti M, Filice C, Bruno A, et al. Long-term sonographic and serological follow-up of inactive echinococcal cysts of the liver: hints for a "watch-and-wait" approach. PLoS Negl Trop Dis. 2014;8:e3057. 10.1371/journal.pntd.0003057 25122222 PMC4133254

[28] Tamarozzi F, Covini I, Mariconti M, Narra R, Tinelli C, De Silvestri A, et al. Comparison of the diagnostic accuracy of three rapid tests for the serodiagnosis of hepatic cystic echinococcosis in humans. PLoS Negl Trop Dis. 2016;10:e0004444. 10.1371/journal.pntd.0004444 26871432 PMC4752287

[29] Pagnozzi D, Tamarozzi F, Roggio AM, Tedde V, Addis MF, Pisanu S, et al. Structural and immunodiagnostic characterization of synthetic antigen B subunits from Echinococcus granulosus and their evaluation as target antigens for cyst viability assessment. Clin Infect Dis. 2018;66:1342–1351. 10.1093/cid/cix1006 29149256 PMC5905600

[30] Han X, Kim JG, Wang H, Cai H, Ma X, Duong DH, et al. Survey of echinococcoses in southeastern Qinghai Province, China, and serodiagnostic insights of recombinant Echinococcus granulosus antigen B isoforms. Parasit Vectors. 2019;12:323. 10.1186/s13071-019-3569-6 31242932 PMC6593596

[31] Gavidia CM, Gonzalez AE, Zhang W, McManus DP, Lopera L, Ninaquispe B, et al. Diagnosis of cystic echinococcosis, central Peruvian Highlands. Emerg Infect Dis. 2008;14:260–166. 10.3201/eid1402.061101 18258119 PMC2600205

[32] Li J, Zhang WB, Wilson M, Ito A, McManus DP. A novel recombinant antigen for immunodiagnosis of human cystic echinococcosis. J Infect Dis. 2003;188:1951–1960. 10.1086/379976 14673776

