# Supplementary material for: Serology for the diagnosis of human hepatic cystic echinococcosis and its relation with cyst staging: A systematic review of the literature with meta-analysis
Source: PLoS Negl Trop Dis. 2021 Apr 28;15(4):e0009370. doi: 10.1371/journal.pntd.0009370 (PMC8081258; doi:10.1371/journal.pntd.0009370)
Supplement: S1 Text — (DOCX) [file pntd.0009370.s002.docx]

**Electronic searches**

A literature search was carried out to identify all possible studies that could help to answer the investigational questions. The following databases were searched for relevant studies:

- Cochrane Central Register of Controlled Trials (CENTRAL 2019, Issue 9);
- MEDLINE (PubMed) (1966 to 2 ottobre 2019);
- EMBASE (Embase.com) (1974 to 2 ottobre 2019);
- Latin American and Caribbean Health Science Information Database (LILACS) (Bireme) (1982 to 2 ottobre 2019).

**Search Strategy**

**MEDLINE (PubMed)**

((((((((((((((((((((((((("Echinococcosis, Pulmonary"[Mesh]) OR "Alveolar echinococcosis"[Supplementary Concept])) OR ("Echinococcosis, Hepatic"[Mesh]) OR (("Echinococcus"[Mesh]))) OR "Echinococcosis"[Mesh])) OR ((((Echinococcoses[Title/Abstract] OR "Echinococcus Infection*"[Title/Abstract] OR "Cystic Echinococcosis"[Title/Abstract] OR "Cystic Echinococcoses"[Title/Abstract] OR Hydatidosis[Title/Abstract] OR Hydatidoses[Title/Abstract] OR "Hydatid Cyst"[Title/Abstract] OR "Hydatid Cysts"[Title/Abstract] OR "Hydatid Disease"[Title/Abstract] OR "Hydatid Diseases"[Title/Abstract] OR "Echinococcus Granulosus Infection"[Title/Abstract] OR "Echinococcus Granulosus Infections"[Title/Abstract] OR "Granulosus Infection, Echinococcus"[Title/Abstract] OR "Infection Echinococcus Granulosus"[Title/Abstract] OR "Pulmonary Hydatidosis"[Title/Abstract] OR "Pulmonary Echinococcoses"[Title/Abstract] OR "Pulmonary Echinococcosis"[Title/Abstract] OR "Pulmonary Hydatid Cyst"[Title/Abstract] OR "Pulmonary Hydatid Cysts"[Title/Abstract] OR "Pulmonary Hydatidoses"[Title/Abstract] OR Alveolococcosis[Title/Abstract] OR "Echinococcus Multilocularis Infection"[Title/Abstract] OR "Alveolar hydatid disease"[Title/Abstract] OR "Multilocular echinococcosis"[Title/Abstract]))))) OR (("Hydatid Liver"[Title/Abstract] OR "Hydatid Liver disease" [Title/Abstract] OR "Hepatic Hydatid Cyst"[Title/Abstract])))))))))))

AND

((((((("Serologic Tests"[Mesh]) OR "Serology"[Mesh])) OR (("Serological Test"[Title/Abstract] OR "Serological Tests"[Title/Abstract] OR "Test, Serological"[Title/Abstract] OR "Tests, Serological"[Title/Abstract] OR "Test, Serologic"[Title/Abstract] OR "Tests, Serologic"[Title/Abstract] OR "Serologic Test"[Title/Abstract] OR "Serologic Tests"[Title/Abstract] OR Serodiagnosis[Title/Abstract] OR Serodiagnoses[Title/Abstract] OR serology[Title/Abstract])))))) OR ((“serological investigations”[Title/Abstract]

**EMBASE (Embase.com)**

#1 'echinococcosis'/exp/mj

#2 echinococcoses OR 'echinococcus infection' OR 'cystic echinococcosis' OR 'cystic echinococcoses' OR hydatidosis OR hydatidoses OR 'hydatid cyst' OR 'hydatid cysts' OR 'hydatid disease' OR 'hydatid diseases' OR 'echinococcus granulosus infection' OR 'echinococcus granulosus infections' OR 'granulosus infection, echinococcus' OR 'infection echinococcus granulosus' OR 'pulmonary hydatidosis' OR 'pulmonary echinococcoses' OR 'pulmonary echinococcosis' OR 'pulmonary hydatid cyst' OR 'pulmonary hydatid cysts' OR 'pulmonary hydatidoses' OR alveolococcosis OR 'echinococcus multilocularis infection' OR 'alveolar hydatid disease' OR 'multilocular echinococcosis' OR 'hydatid liver' OR 'hydatid liver disease' OR 'hepatic hydatid cyst':de,ti,ab

#3 #1 OR #2

#4 'serology'/exp/mj

#5 'serological test' OR 'serological tests' OR 'test, serological' OR 'tests, serological' OR 'test, serologic' OR 'tests, serologic' OR 'serologic test' OR 'serologic tests' OR serodiagnosis OR serodiagnoses OR serology OR ‘serological investigations’:de,ti,ab

#6 #4 OR #5

#7 #3 AND #6 AND [embase]/lim

**CENTRAL (the Cochrane Library) and LILACS (BIREME) (Latin American and Caribbean Health Science Information database)**

We used below free-text terms combined with MeSH

Echinococcosis OR Echinococcos* OR “Echinococcus Infection*” OR “Hydatid Cyst*” OR “Cystic Echinocccos*” AND 'serological test' OR ‘serological investigations’
