# Supplementary material for: Serology for the diagnosis of human hepatic cystic echinococcosis and its relation with cyst staging: A systematic review of the literature with meta-analysis
Source: PLoS Negl Trop Dis. 2021 Apr 28;15(4):e0009370. doi: 10.1371/journal.pntd.0009370 (PMC8081258; doi:10.1371/journal.pntd.0009370)
Supplement: S2 Text — (DOCX) [file pntd.0009370.s003.docx]

**Adapted version of the Newcastle-Ottawa Quality Assessment Scale used to rank the quality of the studies included in the meta-analysis.**

| **Domains** | **Items** |
| --- | --- |
| **Selection** | 1. Representativeness of the cases 2. Consecutive or obviously representative series of cases 🟏 3. Potential for selection biases or not stated |
|  | 1. Case definition based on imaging 2. Based on ultrasonographu 🟏 3. Based on other imaging techniques |
|  | 1. Staging system of CL lesions adequate   a. CL clearly not included as CE🟏  b. CL included or not clearly excluded from CE group |
|  | 1. Patients classification description   a. Classification of patients in one defined cyst stage group when multiple lesions are present clearly described🟏  b. Classification of patients in one defined cyst stage group when multiple lesions are present not clearly described |
|  | 1. Previous treatments   a. Clearly indicated 🟏  b. Not clearly indicated |
| **Comparability** | 1. Grouping of cyst stages for analysis   a. Yes  b. No🟏 |
| **Outcome** | 1. Blind reading of serological results in respect to imaging   a. Yes 🟏  b. No  c. Not stated |

**Adapted version of the Newcastle-Ottawa Quality Assessment Scale used to rank the quality of the studies included in the meta-analysis. Low quality: studies awarded 1 to 3 stars; high quality: studies awarded 4 or 5 stars; very high quality: studies awarded 6 or 7 stars.**
