# Supplementary figures and images for: Serology for the diagnosis of human hepatic cystic echinococcosis and its relation with cyst staging: A systematic review of the literature with meta-analysis
Source: PLoS Negl Trop Dis. 2021 Apr 28;15(4):e0009370. doi: 10.1371/journal.pntd.0009370 (PMC8081258; doi:10.1371/journal.pntd.0009370)

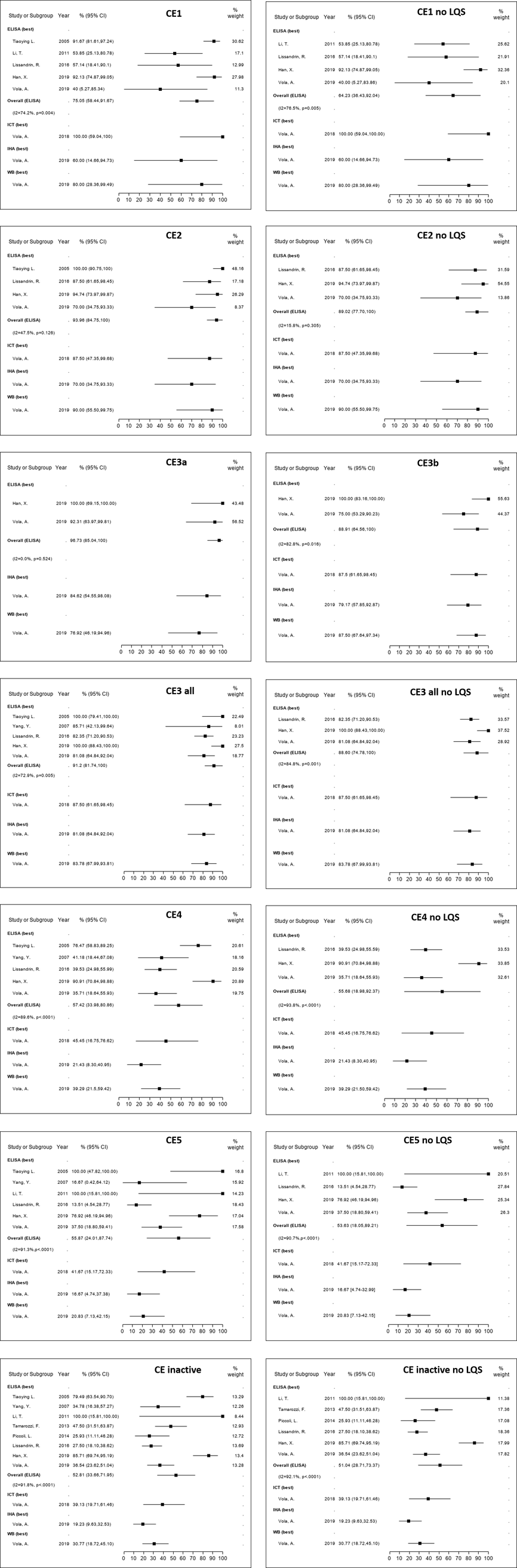

Supplement: S1 Fig — (TIF) [file pntd.0009370.s006.tif]

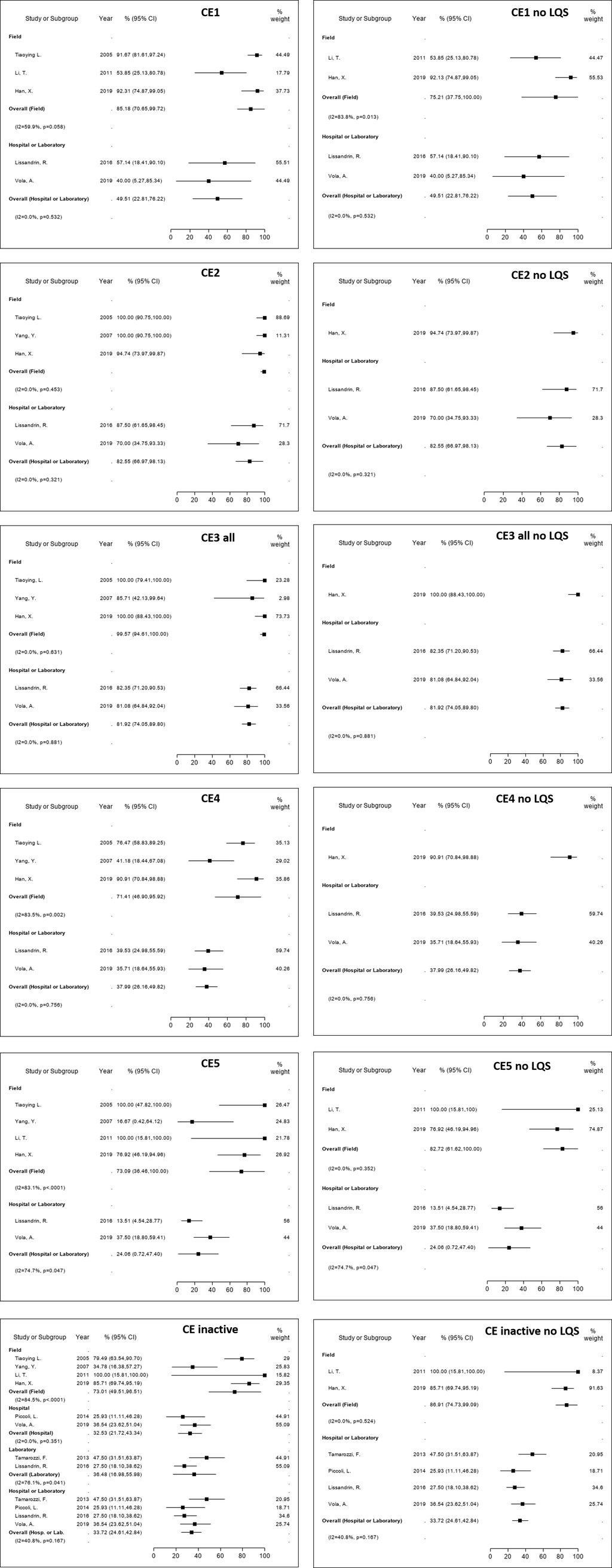

Supplement: S2 Fig — (TIF) [file pntd.0009370.s007.tif]

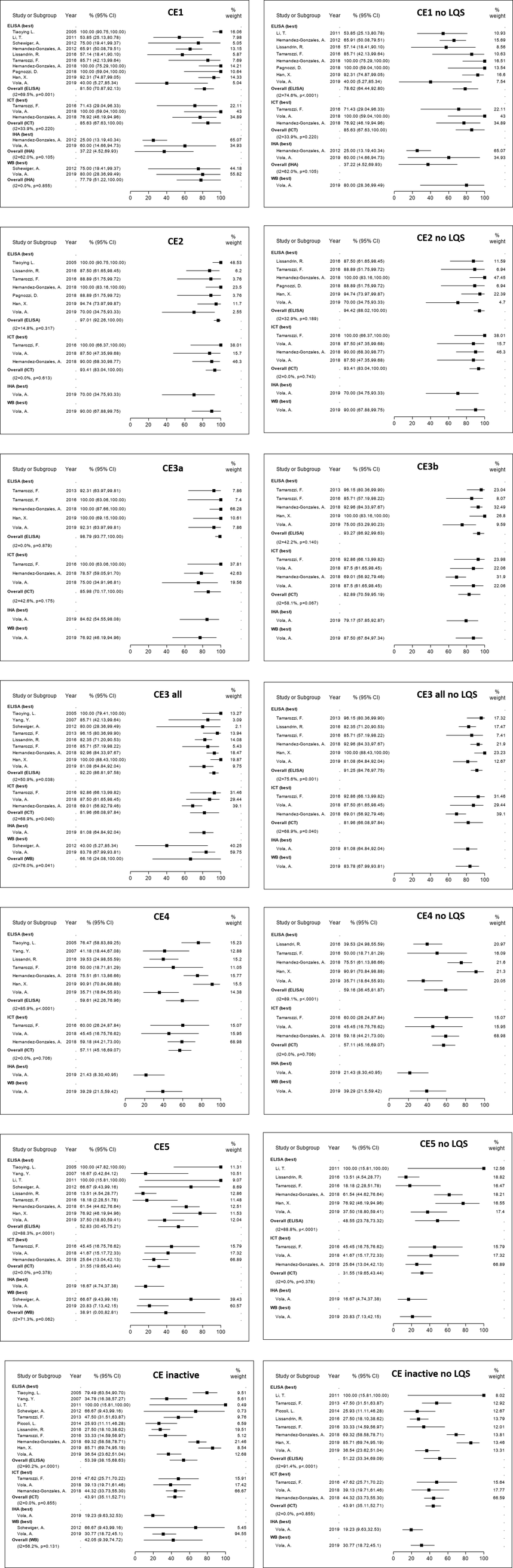

Supplement: S3 Fig — (TIF) [file pntd.0009370.s008.tif]
