# Supplementary material for: Serology for the diagnosis of human hepatic cystic echinococcosis and its relation with cyst staging: A systematic review of the literature with meta-analysis
Source: PLoS Negl Trop Dis. 2021 Apr 28;15(4):e0009370. doi: 10.1371/journal.pntd.0009370 (PMC8081258; doi:10.1371/journal.pntd.0009370)
Supplement: Supplementary file 6 [file pntd.0009370.s004.docx]

Firts author	Year	Journal	SELECTION					COMPARABILITY	OUTCOME		
			Representativeness	Case definition	CL	Patients classification	Treatments	Grouping of cyst stages	Blinding	TOTAL* (n)	CLASSIFICATION
**Only not-treated cysts**											
Tiaoying, L.	2005	Emerg Infect Dis	*	*						** (2)	Low Quality
Yang, Y	2007	Trop Med Int Health	*	*			*			*** (3)	Low Quality
Li, T.	2011	PLoS Negl Trop Dis	*	*			*	*		**** (4)	High Quality
Tamarozzi, F.	2013	Parasite Immunol		*	*	*	*		*	***** (5)	High Quality
Piccoli, L.	2014	PLoS Negl Trop Dis	*	*	*	*	*			***** (5)	High Quality
Vola, A.	2018	Diagn Microbiol Infect Dis		*	*	*		*	*	***** (5)	High Quality
Han, X.	2019	Parasit vectprs	*	*	*	*	*	*		****** (6)	Very High Quality
Vola, A.	2019	Am J Trop Med Hyg	*	*	*	*	*	*		****** (6)	Very High Quality
Lissandrin, R.	2016	Am J Trop Med Hyg	*	*	*	*	*	*		****** (6)	Very High Quality
**Treated and not-treated cysts**											
Schewiger, A.	2012	Infection		*	*					** (2)	Low Quality
Hernandez-Gonzalez, A.	2012	PLoS Negl Trop Dis		*	*		*	*		**** (4)	High Quality
Tamarozzi, F.	2013	Parasite Immunol	See above								
Tamarozzi, F	2016	PLoS Negl Trop Dis		*	*	*	*	*	*	****** (6)	Very High Quality
Hernandez-Gonzalez, A.	2018	PLoS Negl Trop Dis	*	*	*	*		*		***** (5)	High Quality
Pagnozzi, D.	2018	Clin Infect Dis		*	*		*			*** (3)	Low Quality
Vola, A.	2018	Diagn Microbiol Infect Dis	See above								
